# A 23‐Gene Classifier urine test for prostate cancer prognosis

**DOI:** 10.1002/ctm2.340

**Published:** 2021-03-01

**Authors:** Jinan Guo, Heather Johnson, Xuhui Zhang, Xiaoyan Feng, Heqiu Zhang, Athanasios Simoulis, Alan HB Wu, Taolin Xia, Fei Li, Wanlong Tan, Allan Johnson, Nishtman Dizeyi, Per‐Anders Abrahamsson, Lukas Kenner, Lingwu Chen, Wanmei Zhong, Kefeng Xiao, Jenny L. Persson, Chang Zou

**Affiliations:** ^1^ Shenzhen People’s Hospital (The Second Clinical Medical College, Jinan University; The First Affiliated Hospital, Southern University of Science and Technology), Shenzhen Urology Minimally Invasive Engineering Centre Shenzhen China; ^2^ Shenzhen Public Service Platform on Tumor Precision Medicine and Molecular Diagnosis, Clinical Medical Research Centre Shenzhen China; ^3^ Olympia Diagnostics, Inc. Sunnyvale California; ^4^ Department of Bio‐Diagnosis Institute of Basic Medical Sciences Beijing China; ^5^ Department of Clinical Pathology and Cytology Skåne University Hospital Malmö Sweden; ^6^ Clinical Laboratories San Francisco General Hospital San Francisco California; ^7^ Department of Urology Foshan First People's Hospital Foshan China; ^8^ Department of Urology, Nanfang Hospital Southern Medical University Guangzhou China; ^9^ Kinetic Reality Santa Clara California; ^10^ Department of Translational Medicine Lund University, Clinical Research Centre Malmö Sweden; ^11^ Department of Experimental Pathology, Medical University Vienna & Unit of Laboratory Animal Pathology University of Veterinary Medicine Vienna Austria; ^12^ Department of Urology The First Affiliated Hospital of Sun Yat‐Sen University Guangzhou China; ^13^ Department of Molecular Biology Umeå University Umeå Sweden; ^14^ Department of Biomedical Sciences Malmö University Malmö Sweden; ^15^ Division of Experimental Cancer Research, Department of Translational Medicine Lund University Malmö Sweden

Dear Editor,

Currently no accurate prognostic test is available to predict prostate cancer (PCa) biochemical recurrence (BCR) after treatment or cancer metastasis.^1^, ^2^, ^3^, ^4^, ^5^, ^6^, ^7^ To address the unmet medical need, we developed a novel 23‐Gene Classifier urine test as the first accurate and noninvasive tool for PCa prognosis with potential to improve cancer treatment.

We used previously identified biomarkers with differential gene expression in PCa and benign prostate as candidates for BCR prediction and metastasis.^8^, ^9^, ^10^ Discriminant analysis was used to assess the ability of various combinations of mRNA expression quantities of the biomarker candidates in prostate tissue specimens collected before prostatectomy with BCR information during follow‐up as classifiers to distinguish BCR and non‐BCR patients. A 23‐Gene Classifier consisting of *PTEN, PIP5K1A, CDK1, TMPRSS2, ANXA3, HIF1A, FGFR1, BIRC5, AMACR, CRISP3, PMP22, GOLPH2, EZH2, GSTP1, PCA3, VEGFA, CST3, CCNA1, CCND1, FN1, MYO6, KLK3*, and *PSCA* was found to predict BCR with the highest accuracy. We followed STARD guidelines for biomarker validation. Detailed patient cohorts and study methods are described in Supplementary Methods.

The prostate epithelial cells are released into the urine so urine can be used as a noninvasive liquid biopsy source to detect prostate‐specific biomarkers for PCa prognosis. The 23‐Gene Classifier was developed as a urine test for BCR prognosis using urines collected without digital rectal examination (DRE). Using BCR Urine Prediction Algorithm, the mRNA levels of the 23 genes were used to generate a classification score to predict the patients as having BCR or Non‐BCR (Supplementary Methods). A multicenter study was designed prospectively using retrospectively collected urine samples without DRE from 520 patients before prostatectomy or other treatments (IND‐CHTN cohort). Forty‐six patients developed BCR during the follow‐up period averaging 8 years (Table 1). A total of 105 patients from the cohort were randomly selected as a training set to test the 23‐Gene Classifier urine test for BCR prediction and the resulting area under the receiver operating characteristic curve (AUC) was 0.94 (95% CI 0.87‐1.01).

**TABLE 1:** Patient characteristics

	MSKCC cohort	IND‐CHTN cohort	7‐HOSPITALS cohort
No. of patients	150	520	207
Mean age (year range)	58 (37‐79)	63 (43‐78)	69 (39‐88)
No. of Gleason score (%)			
Group 1: ≤6 (≤3+3)	41 (27.33%)	122 (23.46%)	42 (20.29%)
Group 2: 7 (3+4)	53 (35.33%)	220 (42.31%)	57 (27.54%)
Group 3: 7 (4+3)	24 (16.00%)	138 (26.54%)	42 (20.29%)
Group 4: 8 (4+4, 3+5, 5+3)	11 (7.33%)	14 (2.69%)	35 (16.91%)
Group 5: 9 or 10 (4+5, 5+4, or 5+5)	10 (6.70%)	25 (4.80%)	31 (14.98%)
Unknown	11 (7.30%)	1 (0.20%)	0
No. of PSA (ng/dL)			
PSA < 10 ng/dL (%)	115 (76.67%)	0	65 (31.40%)
PSA 10‐20 ng/dL (%)	18 (12.00%)	0	49 (23.67%)
PSA > 20 ng/dL (%)	14 (9.33%)	0	91 (43.96%)
PSA unknown (%)	3 (2.00%)	520 (100%)	2 (0.97%)
Distant metastasis (%)	19 (12.67%)	8 (1.54%)	51 (24.64%)
Bone Met (%)	2 (1.33%)	6 (1.15%)	32 (15.46%)
Other sites Met (%)	17 (11.33%)	2 (0.38%)	26 (12.56%)
Biochemical recurrence (%)	36 (24.00%)	46 (8.85%)	0

PSA: prostate specific antigen; Met: cancer metastasis.

The prognostic performance of the 23‐Gene Classifier urine test to predict BCR‐free survival was validated in the remaining patients (*n* = 414). The patients were divided into two risk groups based on diagnosis by the 23‐Gene Classifier and Kaplan‐Meier survival analysis showed statistically significant association of the 23‐Gene Classifier Negative group with shorter BCR‐free survival (∼60% BCR‐free survival at 48 months) as compared with the 23‐Gene Classifier Positive group (100% BCR‐free survival at 120 months) (Figure 1A) (log rank *P *= 0.000). In contrast, the two groups segregated by cancer stage or Gleason score had much smaller difference in BCR‐free survival (Figures 1B and C).

FIGURE 1Kaplan‐Meier survival curves and receiver operating characteristic (ROC) curves of the 23‐Gene Classifier, Gleason score and cancer stage for prediction of BCR‐free survival in the IND‐CHTN urine study cohort and the MSKCC tissue cohort. Kaplan‐Meier survival curve of the 23‐Gene Classifier (A) (log rank *P* = 0.000), Gleason score (B) (log rank *P* = 0.137), and cancer stage (C) (log rank *P* = 0.013) in the IND‐CHTN cohort. Kaplan‐Meier survival curve of the 23‐Gene Classifier (D) (log rank *P* = 0.000), Gleason score (E) (log rank *P* = 0.001), and cancer stage (F) (log rank *P* = 0.000) in the MSKCC cohort. ROC curve of the 23‐Gene Classifier (G), cancer stage (H), Gleason score (I), and combination of the 23‐Gene Classifier, cancer stage and Gleason score (J) for BCR prediction in the IND‐CHTN cohort. ROC curve of the 23‐Gene Classifier (K), cancer stage (L), Gleason score (M), and combination of the 23‐Gene Classifier, cancer stage and Gleason score (N) for BCR prediction in the MSKCC cohort

**Figure:**
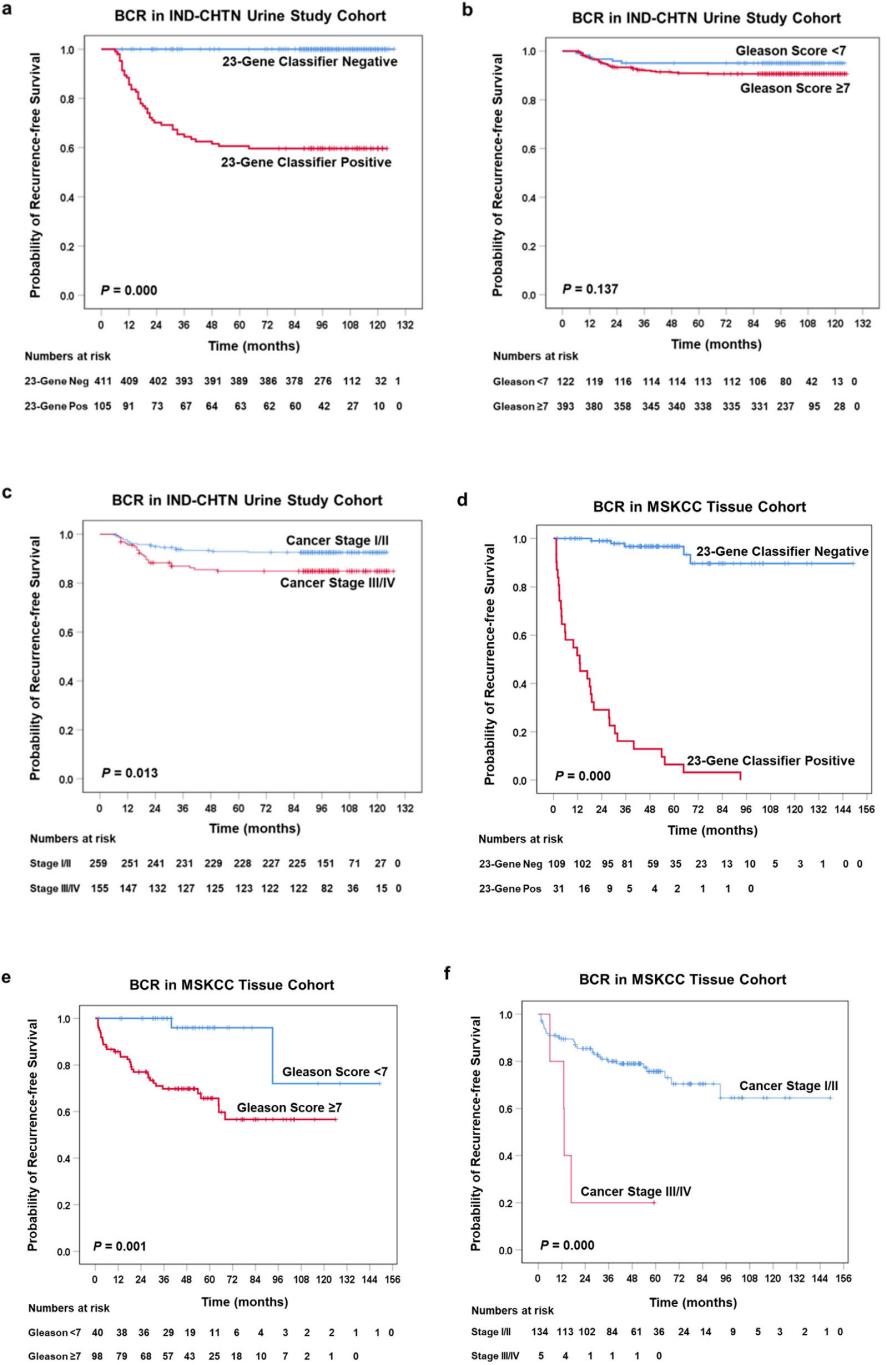

**Figure:**
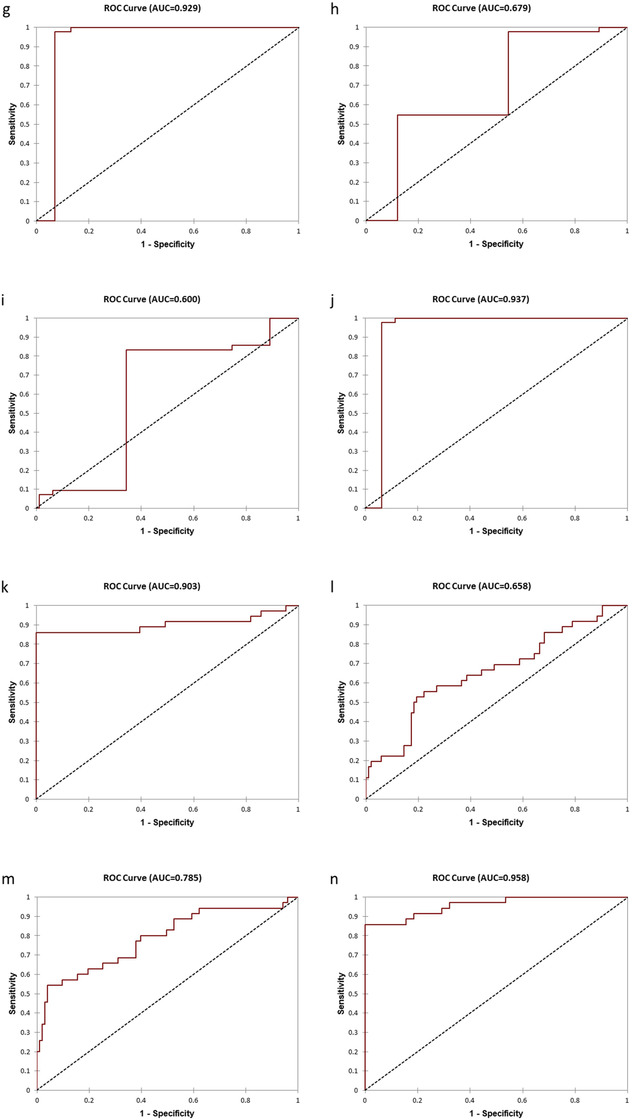

Univariate and multivariate Cox regression analysis was performed and the 23‐Gene Classifier had a hazard ratio (HR) of 1730.90 (95% CI 4.52‐6.63E+5) in the univariate analysis (Table 2), which indicated that the patients with a positive 23‐Gene Classifier score was 1731 times more likely to have BCR than patients with a negative 23‐Gene Classifier score and the BCR prediction was statistically significant (*P *= 0.014). Its predictive power remained large and significant in multivariate regression after adjusting for cancer stage and Gleason score with HR of 1795.01 (95% CI 4.30‐7.49E+5) (*P *= 0.015). In contrast, cancer stage and Gleason score had much lower HR and were statistically insignificant (Table 2).

**TABLE 2:** Cox regression analysis of BCR‐free survival using the 23‐Gene Classifier, cancer stage, and Gleason score in IND‐CHTN urine study cohort and MSKCC tissue cohort

Variable	Univariate			Multivariate	
	HR (95% CI)		*P* value	HR (95% CI)	*P* value
**IND‐CHTN cohort (*n* = 414)**					
Cancer stage	22.19 (0.09‐5.34E+3)		0.268	10.11 (0.05‐2.21E+3)	0.400
Gleason score	20.76 (0.00‐2.17E+5)		0.521	106.03 (0.00‐2.74E+7)	0.463
23G classifier	1730.90 (4.52‐6.63E+5)		0.014	1795.01 (4.30‐7.49E+5)	0.015
**MSKCC cohort (*n* = 140)**					
Cancer stage	5.21 (2.14‐12.68)	0.000		0.52 (0.20‐1.37)	0.186
Gleason score	11.59 (5.84‐23.01)	0.000		2.864 (1.30‐6.30)	0.009
23G classifier	54.23 (20.67‐142.24)	0.000		44.01 (15.91‐121.75)	0.000

HR: hazard ratio; CI: confidence interval; 23G Classifier: 23‐Gene Classifier.

In addition, univariate and multivariate logistic regression and discriminant analysis were performed to measure the predictive accuracy of the 23‐Gene Classifier. The result showed high accuracy with sensitivity of 100% (95% CI 100‐100%), specificity of 86.29% (95% CI 82.80‐89.79%), and AUC of 0.93 (95% CI 0.90‐0.96) (*P *< 0.0001) (Tables S1 and 3, Figure 1G). Cross‐validation of the 23‐Gene Classifier showed similarly high accuracy in BCR prediction (Table 3). In contrast, cancer stage and Gleason score had much lower specificity and AUC (Table 3, Figures 1H and I).

**TABLE 3:** Prognostic performance of the 23‐Gene Classifier, cancer stage, Gleason score, and their combination for BCR prediction in IND‐CHTN urine study and MSKCC prostate tissue cohorts

	Sensitivity (95% CI)	Specificity (95% CI)	PPV (95% CI)	NPV (95% CI)	AUC (95% CI)
IND‐CHTN cohort (*n* = 414)					
Cancer stage	100% (100‐100%)	7.28% (4.63‐9.92%)	10.88% (7.77‐13.99%)	100% (100‐100%)	0.68 (0.60‐0.76)
Gleason score	100% (100‐100%)	2.43% (0.86‐3.99%)	10.40% (7.42‐13.37%)	100% (100‐100%)	0.60 (0.52‐0.69)
23G classifier	100% (100‐100%)	86.29% (82.80‐89.79%)	45.16% (35.05‐55.28%)	100% (100‐100%)	0.93 (0.90‐0.96)
23G classifier Cross‐validation	100% (100‐100%)	86.17% (82.14‐90.20%)	45.07% (33.50‐56.64%)	100% (100‐100%)	0.93 (0.90‐0.96)
Combination	100% (100‐100%)	88.11% (84.81‐91.41%)	48.84% (38.27‐59.40%)	100% (100‐100%)	0.94 (0.91‐0.96)
MSKCC Cohort (*n* = 140)					
Cancer stage	16.67% (4.49‐28.84%)	99.04% (97.16‐100.91%)	85.71% (59.79‐111.64%)	77.44% (70.34‐84.55%)	0.66 (0.56‐0.76)
Gleason Score	48.57% (32.01‐65.13%)	96.12% (92.39‐99.85%)	80.95% (64.16‐97.75%)	84.62% (78.08‐91.15%)	0.79 (0.71‐0.86)
23G classifier	86.11% (74.81‐97.41%)	100% (100‐100%)	100% (100‐100%)	95.41% (91.49‐99.34%)	0.90 (0.85‐0.95)
23G classifier Cross‐validation	87.50% (64.58‐110.42%)	100% (100‐100%)	100% (100‐100%)	96.97% (91.12‐102.82%)	0.88 (0.78‐0.99)
Combination	85.71% (74.12‐97.31%)	100% (100‐100%)	100% (100‐100%)	95.37% (91.41‐99.33%)	0.96 (0.93‐0.99)

AUC: area under the ROC curve; CI: confidence interval; PPV: positive predictive value; NPV: negative predictive value; 23G Classifier: 23‐Gene Classifier; Combination: combining cancer stage, Gleason score, and 23‐Gene Classifier.

*In silico* validation study was conducted to test if the 23‐Gene Classifier can also be used in prostate tissue specimens for BCR prognosis using a tissue cohort MSKCC (Table 1). Its similarly high prognostic performance (Tables 2 and 3, Figures 1D‐F, K‐N) validated the results from the urine study and confirmed the 23‐Gene Classifier as a more accurate prognostic tool for BCR prediction than cancer stage and Gleason score.

Accurate prediction of cancer metastasis at diagnosis is important for patients to be treated early with effective therapies to prevent development of castration‐resistant metastatic cancer and reduce mortality. We tested if the 23‐Gene Classifier urine test could be used for metastatic cancer prediction. We tested its performance in the multicenter, retrospective IND‐CHTN Cohort (*n* = 520), a multicenter, prospective 7‐HOSPITALS Cohort (*n* = 207), and a combination cohort combining the patients (*n* = 727) (Table 1). mRNA expression quantities of the 23 genes were used to classifier each sample as metastatic or nonmetastatic cancer using MET Urine Prediction Algorithm and such classification was compared with the metastatic cancer diagnosis by the imaging measurements to calculate the predictive performance (Supplementary Methods). The result showed that the 23‐Gene Classifier urine test had similarly high accuracy in predicting metastatic cancer in the retrospective, prospective and combination cohorts (AUC of 0.92 [95% CI 0.79‐1.05] for the retrospective cohort, 0.89 [95% CI 0.83‐0.95] for the prospective cohort, and 0.98 [95% CI 0.96‐1.01] for the combination cohort) (*P *< 0.0001). In contrast, Gleason score had much lower specificity and AUC (Table S2 and Figure 2).

FIGURE 2Receiver operating characteristic (ROC) curves of the 23‐Gene Classifier for prediction of metastatic cancer in the urine cohorts. ROC curve of the 23‐Gene Classifier (A), Gleason score (B), and combination of the 23‐Gene Classifier and Gleason score (C) in the retrospective urine cohort. ROC curve of the 23‐Gene Classifier (D), Gleason score (E), and combination of the 23‐Gene Classifier and Gleason score (F) in the prospective urine cohort. ROC curve of the 23‐Gene Classifier (G), Gleason score (H), and combination of the 23‐Gene Classifier and Gleason score (I) in the combination urine cohort

**Figure:**
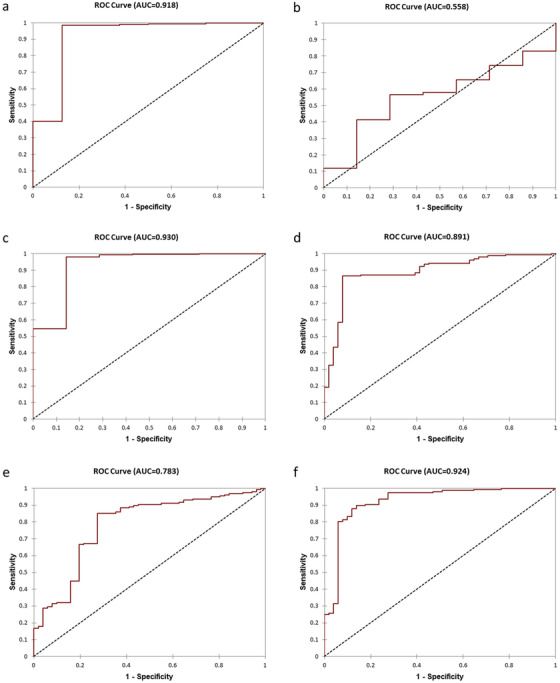

**Figure:**
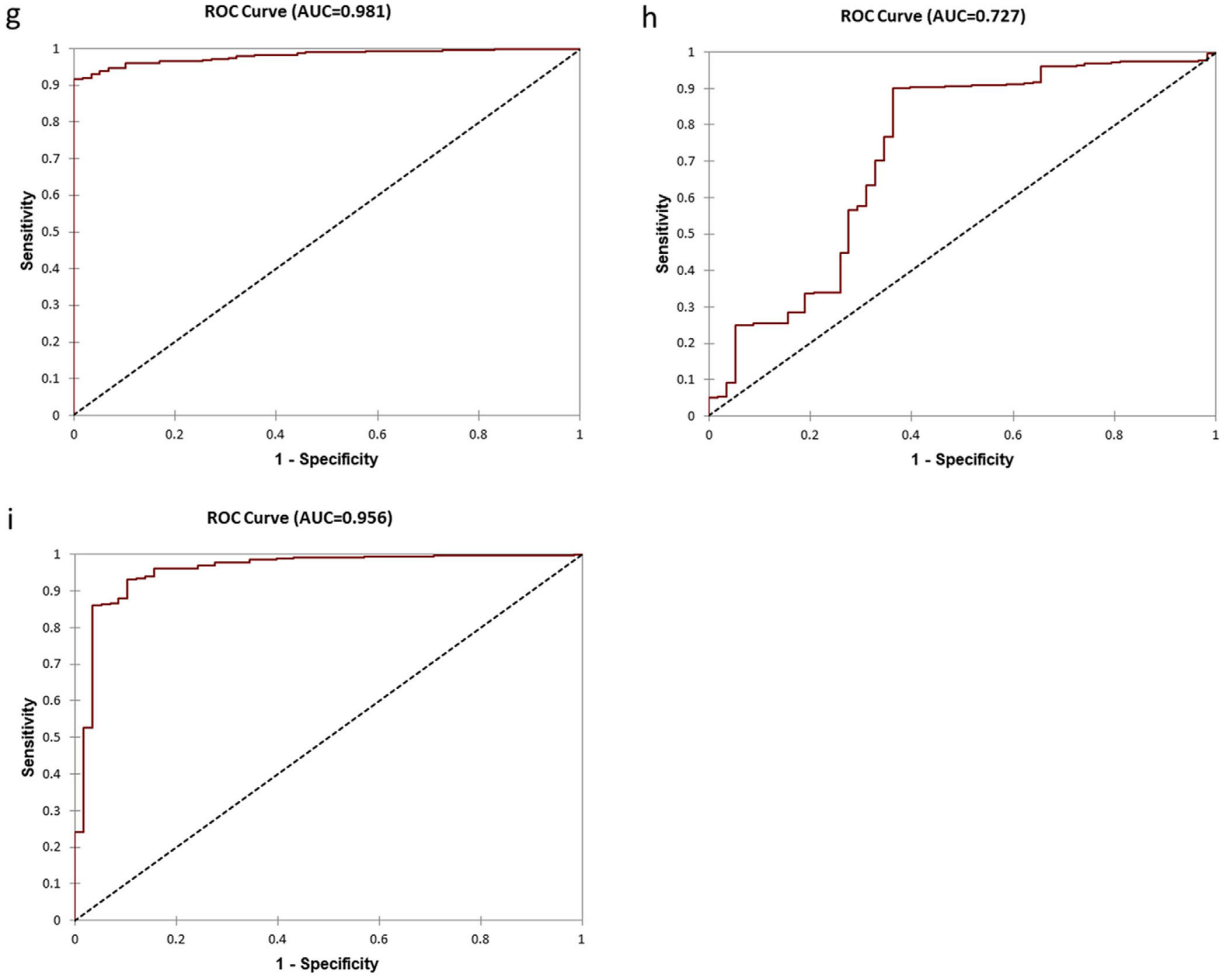

Development of accurate and actionable prognostic tests is important and urgently needed for PCa treatment. None of the clinicopathological parameters, nomograms, or biomarker panels used in clinic or reported in publications was capable of accurately predicting BCR or cancer metastasis with HR above 20 or AUC above 0.9.^1^, ^2^, ^3^, ^4^, ^5^, ^6^, ^7^ The 23‐Gene Classifier had HR above 40 and AUC above 0.9 in all cohorts assessed, suggesting its higher accuracy and more robust performance for PCa prognosis. In addition, the 23‐Gene Classifier can be used with prostate tissue specimens.

In this study, we developed and validated a novel 23‐Gene Classifier that can be used as a highly accurate and noninvasive urine test for prediction of BCR and cancer metastasis with great potential to improve PCa treatment and reduce mortality in clinical practice.

## ETHICS APPROVAL AND CONSENT TO PARTICIPATE

The retrospective urine study was approved by IRB at San Francisco General Hospital (IRB #: 15–15816) to use archived urine sediment samples acquired from Cooperative Human Tissue Network Southern Division and Indivumed GmbH. These organizations obtained ethical approval and patient consent prior to collection of patient urine samples. The prospective urine study was approved by IRB at Shenzhen People's Hospital (Study Number: P2014‐006) to use urine samples collected from patients treated at the collaborating hospitals in the study with prior consent.

## CONSENT FOR PUBLICATION

All authors have agreed to publish the manuscript.

## DATA AVAILABILITY STATEMENT

The data supporting this study are available from the corresponding authors upon reasonable request or are publicly available in GEO.

## CONFLICT OF INTEREST

Heather Johnson is an employee of Olympia Diagnostics, Inc., and inventor of a pending patent application of prostate cancer diagnostic and prognostic biomarkers. No conflict of interest or financial interest was declared by the other authors.

## FUNDING

This study was supported by grants from Sanming Project of Medicine in Shenzhen (SZSM201412014), The Science and Technology Foundation of Shenzhen (JCYJ20170307095620828), The Science and Technology Foundation of Shenzhen (JCYJ20160422145718224), and The Shenzhen Urology Minimally Invasive Engineering Center (GCZX2015043016165448) (to Jinan Guo, and Kefeng Xiao); funds from Olympia Diagnostics, Inc. (to Heather Johnson); the Swedish Cancer Society (CAN2017/381), The Swedish Children Foundation (TJ2015‐0097), H2020‐MSCA‐ITN‐2018 GlycoImaging (721279), The Swedish National Research Council, the Malmö Cancer Foundation, the Government Health Innovation Grant, the Medical Faculty, Lund University, Kempestiftelserna, Umeå University, Medical Faculty Grants, the Norland Fund for Cancer Forskning, Insamlings Stiftelsen, Umeå University, Bioteknik medel, the Medical Faculty, Umeå University, Medical Faculty Grants, Umeå University, and grant from Umeå University Center for Microbiology Research (UCMR) and Biofilm Center at Malmö University (to Jenny Persson). The funders had no role in study design, data collection and analysis, decision to publish, or preparation of the manuscript.

## AUTHORS' CONTRIBUTIONS

HJ, CZ, LC, KX, and JLP contributed to study concept and design. HZ, JG, XF, CZ, KX, AHBW, and LC participated in study coordination and supervision. JG, TX, FL, and WT contributed to sample collection. XZ, JG, HJ, HZ, and XF contributed to sample processing and analysis. HZ, HJ, AJ, AS, ND, and JLP contributed to data collection and processing, and statistical analysis. HJ, PA, ND, LK, AS, and JLP contributed to data interpretation. XZ, HJ, and JLP contributed to literature search. JG, HJ, HZ, JLP, and CZ contributed to manuscript writing.

## Supporting information

Figure S1 Study design

Figure S1 Study design

Table S1 Univariate and multivariate logistic regression analyses of the 23‐Gene Classifier, cancer stage, and Gleason score for BCR prediction in IND‐CHTN urine study cohort (*n* = 520) and MSKCC prostate tissue cohort (*n* = 140)

Table S2 Prognostic performance of the 23‐Gene Classifier, Gleason score, and their combination for prediction of metastatic prostate cancer in the urine cohorts (*n* = 727)

Supporting Information
