# Supplementary figures and images for: A 23‐Gene Classifier urine test for prostate cancer prognosis
Source: Clin Transl Med. 2021 Mar 1;11(3):e340. doi: 10.1002/ctm2.340 (PMC7919118; doi:10.1002/ctm2.340)

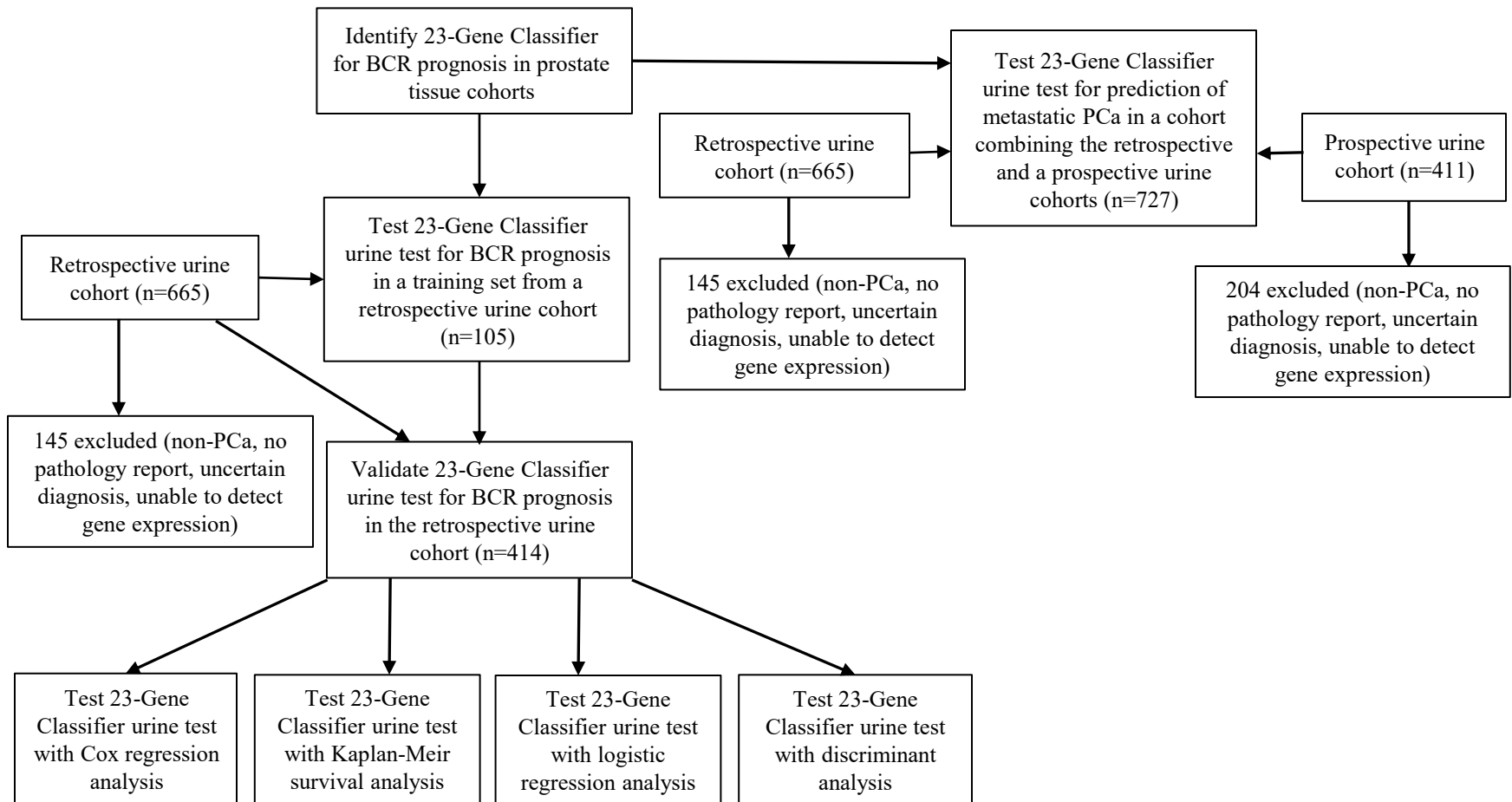

Supplement: Supplementary file 1 — Figure S1 Study design [file CTM2-11-e340-s001.pdf]
