# Supplementary material for: A 23‐Gene Classifier urine test for prostate cancer prognosis
Source: Clin Transl Med. 2021 Mar 1;11(3):e340. doi: 10.1002/ctm2.340 (PMC7919118; doi:10.1002/ctm2.340)
Supplement: Supplementary file 2 — Figure S1 Study design [file CTM2-11-e340-s002.docx]

**Supplementary Figure Legend**

Supplementary Figure S1 Study design.
