# Supplementary material for: A 23‐Gene Classifier urine test for prostate cancer prognosis
Source: Clin Transl Med. 2021 Mar 1;11(3):e340. doi: 10.1002/ctm2.340 (PMC7919118; doi:10.1002/ctm2.340)
Supplement: Supplementary file 3 — Table S1 Univariate and multivariate logistic regression analyses of the 23‐Gene Classifier, cancer stage, and Gleason score for BCR prediction in IND‐CHTN urine study cohort (n = 520) and MSKCC prostate tissue cohort (n = 140) [file CTM2-11-e340-s003.docx]

Supplementary Table S1 Univariate and multivariate logistic regression analyses of the 23-Gene Classifier, cancer stage and Gleason score for BCR prediction in IND-CHTN urine study cohort (n=520) and MSKCC prostate tissue cohort (n=140).

|  | | **IND-CHTN Cohort** | | | | **MSKCC Cohort** | | | |
| --- | --- | --- | --- | --- | --- | --- | --- | --- | --- |
|  |  | **Univariate** | | **Multivariate** | | **Univariate** | | **Multivariate** | |
|  | *P*-value | | AUC (95% CI) | *P*-value | AUC (95% CI) | *P*-value | AUC (95% CI) | *P*-value | AUC (95% CI) |
| Cancer Stage | 0.014 | | 0.68  (0.60-0.75) | <0.0001 | - | 0.327 | 0.67  (0.56-0.76) | 0.881 | - |
| Gleason Score | 0.907 | | 0.60  (0.51-0.68) | 0.169 | - | <0.0001 | 0.79  (0.71-0.86) | 0.568 | - |
| 23G Classifier | <0.0001 | | 0.93  (0.90-0.96) | <0.0001 | - | <0.0001 | 0.90  (0.85-0.95) | <0.0001 | - |
| Combination | - | | - | <0.0001 | 0.96  (0.94-0.98) | - | - | <0.0001 | 0.97  (0.94-0.99) |

AUC: Area under the ROC Curve; CI: confidence interval; 23G Classifier: 23-Gene Classifier.
