# Supplementary material for: A 23‐Gene Classifier urine test for prostate cancer prognosis
Source: Clin Transl Med. 2021 Mar 1;11(3):e340. doi: 10.1002/ctm2.340 (PMC7919118; doi:10.1002/ctm2.340)
Supplement: Supplementary file 4 — Table S2 Prognostic performance of the 23‐Gene Classifier, Gleason score, and their combination for prediction of metastatic prostate cancer in the urine cohorts (n = 727) [file CTM2-11-e340-s004.docx]

Supplementary Table S2 Prognostic performance of the 23-Gene Classifier, Gleason score and their combination for prediction of metastatic prostate cancer in the urine cohorts (n=727).

|  | **Sensitivity**  **(95% CI)** | **Specificity**  **(95% CI)** | **PPV**  **(95% CI)** | **NPV**  **(95% CI)** | **AUC**  **(95% CI)** |
| --- | --- | --- | --- | --- | --- |
| 23G Classifier in  Retrospective Cohort | 87.50%  (64.58-110.42%) | 97.27%  (95.85-98.68%) | 33.33%  (13.17-53.50%) | 99.80%  (99.41-100.19%) | 0.92 (0.79-1.05) |
| Gleason Score in  Retrospective Cohort | 100%  (100-100%) | 9.18%  (6.68-11.68%) | 1.48%  (0.39-2.57%) | 100%  (100-100%) | 0.56 (0.34-0.78) |
| GS+23G Classifier in  Retrospective Cohort | 100%  (100-100%) | 9.18%  (6.68-11.68%) | 1.48%  (0.39-2.57%) | 100%  (100-100%) | 0.93 (0.78-1.01) |
| 23G Classifier in  Prospective Cohort | 90.20%  (82.03-98.36%) | 86.54%  (81.18-91.89%) | 68.66%  (57.55-79.76%) | 96.43%  (93.35-99.50%) | 0.89 (0.83-0.95) |
| Gleason Score in  Prospective Cohort | 72.55%  (60.30-84.80%) | 81.41%  (75.31-87.52%) | 56.06%  (44.09-68.03%) | 90.07%  (85.13-95.01%) | 0.78 (0.70-0.86) |
| GS+23G Classifier in  Prospective Cohort | 86.27%  (76.83-95.72%) | 89.74%  (84.98-94.50%) | 73.33%  (62.14-84.52%) | 95.24%  (91.80-98.68%) | 0.92 (0.87-0.98) |
| 23G Classifier in  Combination Cohort | 89.83%  (82.12-97.54%) | 94.76%  (93.07-96.45%) | 60.23%  (50.00-70.45%) | 99.06%  (98.31-99.81%) | 0.98 (0.96-1.01) |
| Gleason Score in  Combination Cohort | 94.83%  (89.13-100.53%) | 24.10%  (20.86-27.35%) | 90.21%  (87.76-92.67%) | 98.17%  (96.12-100.22%) | 0.73 (0.65-0.80) |
| GS+23G Classifier in  Combination Cohort | 96.55%  (91.86-101.25%) | 85.48%  (82.81-88.15%) | 36.60%  (28.97-44.23%) | 99.65%  (99.17-100.13%) | 0.96 (0.92-0.99) |

AUC: Area under the ROC Curve; CI: confidence interval; PPV: positive predictive value; NPV: negative predictive value; 23G Classifier: 23-Gene Classifier; GS: Gleason score.
