# Supplementary material for: A 23‐Gene Classifier urine test for prostate cancer prognosis
Source: Clin Transl Med. 2021 Mar 1;11(3):e340. doi: 10.1002/ctm2.340 (PMC7919118; doi:10.1002/ctm2.340)
Supplement: Supplementary file 5 — Supporting Information [file CTM2-11-e340-s005.docx]

**Supplementary Methods**

**Retrospective and prospective urine study cohorts**

A multi-center retrospective study was conducted at San Francisco General Hospital (San Francisco, USA) with Institutional Review Board (IRB) approval (IRB #: 15-15816) to test archived urine samples obtained from Cooperative Human Tissue Network (CHTN) Southern Division (patients recruited in the U.S.) and Indivumed GmbH (patients recruited in Germany) with appropriate ethical approval and patient written informed consent before urine collection. The cohort (IND-CHTN) consisted of prospectively designed, retrospectively collected urine samples without prior digital rectal examination (DRE) following Standards for Reporting of Diagnostic Accuracy Studies (STARD) guidelines for biomarker validation. A multi-center prospective study was conducted following STARD guidelines at Shenzhen People’s Hospital (Shenzhen, China) with IRB approval (Study Number: P2014-006) to collect fresh urine samples from patients treated at seven hospitals collaborated in the study with prior written informed consent (7-HOSPITALS Cohort). The urine samples were collected consecutively and prospectively using a standard protocol without DRE. The patient inclusion criteria in both retrospective and prospective studies included age between 18-85, with histopathological diagnosis of PCa, Benign prostatic hyperplasia or prostatitis after urine collection, without treatment of PCa drugs or 5-Alpha Reductase inhibitors before urine collection. The exclusion criteria included having prostatectomy or treatment with PCa drugs or 5-Alpha Reductase inhibitors before urine collection. In the retrospective cohort, all PCa patients who had RP or other treatments were assessed periodically for BCR (defined as consecutive PSA rise above 0.2 ng/mL twice according to National Comprehensive Cancer Network guidelines) during the follow-up period. PCa patients were also assessed periodically for cancer metastasis by imaging with CT, magnetic resonance or X-ray as well as bone scan during the follow-up period. The patients were recruited from July 2004 to November 2014 with follow-up through June, 2015. In the prospective cohort, all PCa patients were assessed for distant metastasis after cancer diagnosis by imaging with CT or magnetic resonance and bone scan. 51 patients in the retrospective cohort (n=665) and 15 in the prospective cohort (n=411) were excluded due to the lack of pathology report, diagnosis uncertainty, or low/no gene expression detected. All samples were de-identified and coded with patient numbers to protect patient privacy following Health Insurance Portability and Accountability Act guidelines.

**Urine processing and gene expression quantification**

The processing of urine samples and acquisition of data were performed without the knowledge of patient clinical information to minimize potential bias. In the retrospective urine study, 10–15 ml urine samples obtained without digital rectal examination (DRE) were pelleted by centrifugation followed by flash-frozen and storage at -80°C for 5–10 years until the start of this study. In the prospective urine study, 15–45 ml urine sample without DRE was collected in a tube containing 5 ml DNA/RNA preservative AssayAssure (Thermo Fisher Scientific, Waltham, MA, USA) or U-Preserve (Hao Rui Jia Biotech Ltd., Beijing, China) and stored at 4°C until being processed within seven days of collection. The urine pellet after centrifugation at 1000×g for 10 min was washed with phosphate-buffered saline (PBS) and then pelleted by a second centrifugation at 1000×g for 10 min. The pellet was processed for RNA purification or immediately frozen on dry ice and stored at -80°C until future RNA purification.

For urine samples in both retrospective and prospective studies, the frozen urine pellet was thawed at 37°C and resuspended in cold PBS followed by centrifugation at 1000 ×g for 10 min. Quick-RNA MicroPrep Kit was used to purify total RNA from the cell pellet following the manufacturer’s procedure (Zymo Research, Irvine, CA, USA). 100 ng purified RNA was used for cDNA reverse transcription using either High Capacity cDNA Reverse Transcription Kit (Life Technologies, Foster City, CA, USA) or iScript Reverse Transcription Supermix for Real Time qRT-PCR (Bio-Rad, Hercules, CA, USA) according to the manufacturers’ protocols. The cDNA from reverse transcription was preamplified using either TaqMan® PreAmp Master Mix (Thermo Fisher Scientific, Waltham, MA, USA) or PCa PreAmplification Mix (Hao Rui Jia Biotech Ltd., Beijing, China) following the manufacturers’ directions. mRNA expression levels of the 23 genes in the classifier were measured by real-time qRT-PCR with predesigned primers and probe assays from Integrated DNA Technologies (San Diego, CA, USA). In the primers/probe assays, the exon-spanning PCR primers were used to detect mRNA expression without the detection of genomic DNA (for *Beta-actin*, Probe: /56-FAM/ CTG CCT CCA CCC ACT CCC A /3IABKFQ/, Forward: AAG TCA GTG TAC AGG TAA GCC, Reverse: GTC CCC CAA CTT GAG ATG TAT G; for *PTEN,* Probe: /5TET/ TCT TCA TAC CAG GAC CAG AGG AAA CCT /3IABKFQ/, Forward: GCT CTA TAC TGC AAA TGC TAT CG, Reverse: CCA CAA ACA GAA CAA GAT GCT; for *PIP5K1A*, Probe: /56-FAM/ GGG AGC CTG AGT ACC AAA CCA GA /3IABKFQ/, Forward: CTC AAC CAC GTA GAA ATC TTG C, Reverse: CCA TCC AGT TAG GCA TTA CCC; for *CDK1*, Probe: /5TET/ ACC CCT TCC TCT TCA CTT TCT AGT CTG AT /3IABKFQ/, Forward: CAT AAG CAC ATC CTG AAG ACT G, Reverse: AAC TAC AGG TCA AGT GGT AGC; for *TMPRSS2*, Probe: /56-FAM/ ACA CGC CAT CAC ACC AGT TAG AGG /3IABKFQ/, Forward: ACA CAC CGA TTC TCG TCC T, Reverse: TGG CCT ACT CTG GAA GTT CA; for *ANXA3*, Probe: /56-FAM/ TGG GCA TTT TGA AGA CTT ACT GTT GGC /3IABKFQ/, Forward: ACT CAT CAG TTC CAA TAC CCT TC, Reverse: TGT GGA CAG CAT AAA AGG AGA; for *HIF1A*, Probe: /5TET/ ACC ATC AGC TAT TTG CGT GTG AGG A /3IABKFQ/, Forward: ATC TGT GCT TTC ATG TCA TCT TC, Reverse: TGA GTT CGC ATC TTG ATA AGG C; for *FGFR1*, Probe: /5TET/ AAC AAA ACA GTG GCC CTG GGT AGC /3IABKFQ/, Forward: ACA CCT TAC ACA TGA ACT CCA C, Reverse: AGC ATC AAC CAC ACA TAC CAG; for *BIRC5*, Probe: /56-FAM / CCC ACT GAG AAC GAG CCA GAC TTG /3IABKFQ/, Forward: TCC TTG AAG CAG AAG AAA CAC T, Reverse: ACC ACC GCA TCT CTA CAT TC; for *AMACR*, Probe: /5TET/ CCC AGA GAT TCT GCA GCG GGA AA /3IABKFQ/, Forward: GCG GTG TCA TGG AGA AAC T, Reverse: CCA CTC AGC CTG GCA TAA ATA; for *CRISP3*, Probe: /5TET/ CTC AGT CCT CTC CCT TGT GAG TCT TCA /3IABKFQ/, Forward: GTT TCC CTT TCC ATA CTC CAC T, Reverse: GGT AAA TAC CCT CCA CTC AAG; for *PMP22*, Probe: /56-FAM/ CAG TTG CGT GTC CAT TGC CCA /3IABKFQ/, Forward: GCT ACA GTT CTG CCA GAG A, Reverse: CTC CTC CTG TTG CTG AGT ATC; for *GOLPH2*, Probe: /56-FAM/ CCT ACG ACC TGA GCC AGT GCA T /3IABKFQ/, Forward: CTT CTA TTC GCT CCT CAC ACT, Reverse: AGA CCA ACC TGG AGA GGA A; for *EZH2*, Probe: /5TET/ CCG GTG TTT CCT CTT CTT TTT CCT TGG A /3IABKFQ/, Forward: TCA GCT GTA TCT TTC TGC AGT G, Reverse: GAC AGG TGT ATG AGT TTA GAG TCA; for *GSTP1*, Probe: /56-FAM/ AGC AGG GTC TCA AAA GGC TTC AGT /3IABKFQ/, Forward: GGT TGT AGT CAG CGA AGG AG, Reverse: GGC AAG GAT GAC TAT GTG AAG G; for *PCA3*, Probe: /56-FAM/ TGC TGA CTT TAC CAT CTG AGG CCA C /3IABKFQ/, Forward: TGT CAT CTT GCT GTT TCT AGT GA, Reverse: GGA AGG ACC TGA TGA TAC AGA G; for *VEGFA*, Probe: /56-FAM/ TCA AAC CTC ACC AAG GCC AGC /3IABKFQ/, Forward: GCT GTA GGA AGC TCA TCT CTC, Reverse: AGT CCA ACA TCA CCA TGC AG; for *CST3*, Probe: /5TET/ TTC ACC CCA GCT ACG ATC TGC TTG /3IABKFQ/, Forward: GCT CCA CGT CCA AGA AGT, Reverse: GAG TAC AAC AAA GCC AGC AAC; for *CCNA1*, Probe: /5TET/ TGC TTC AAG TAG ACT CAG CTC TGC TAC G /3IABKFQ/, Forward: GCC AAA AGT GCT TGT TCA CAG, Reverse: GAG TGT GCG TCA GGA CTG). Real-time qRT-PCR was performed on ABI Quantstudio 6, ABI 7500 or ABI 7900 Real-Time PCR System (Thermo Fisher Scientific, Waltham, MA, USA). Each PCR reaction with 10 μl volume contained preamplified cDNA transcribed from 0.2 ng of purified RNA, 5 μl of 2x TaqMan® Universal PCR Master Mix (Thermo Fisher Scientific, Waltham, MA, USA) or PrimeTime® Gene Expression Master Mix (Integrated DNA Technologies, San Diego, CA, USA), 500 nM each of forward and reverse amplification primers, and 250 nM of probe. The cycling condition of real-time qRT-PCR included: 10 minutes at 95°C for polymerase activation, and 40 cycles of 15 seconds at 95°C and 1 minute at 60°C. Triplicate PCRs were performed for each gene. The gene expression measurement and calculation were performed blindly without patient information. The gene expression data was initially analyzed using ABI Quantstudio 6, ABI 7500 or ABI 7900 software (Thermo Fisher Scientific, Waltham, MA, USA). The mRNA level of a housekeeping gene beta-actin was measured in each urine sample and used to normalize expression of each gene in the classifier to control variation of cDNA quantity in the patient samples. The cycle threshold (Ct) value of each gene in the panels was divided by the Ct value of the beta-actin as the normalized gene expression value (CtS=Ct(sample)/Ct(actin)). For each gene, mean Ct value from triplicate PCRs was used.

**Algorithms for BCR and distant metastasis prediction in urine samples**

For BCR prediction in the urine samples, the relative cycle threshold (CtS) values of the 23 genes in the classifier were used to generate a classification score (BCR Urine P Score) for each sample using the BCR Urine Prediction Algorithm as shown below:

C_BCRU_=A_BCRU_+CtS_1_*BU_1_+CtS_2_*BU_2…_+CtS_23_*BU_23_+CtS_1_*CtS_1_*BU_1*1_+CtS_1_*CtS_2_*BU_1*2…_+CtS_1_*CtS_23_*BU_1*23_+CtS_2_*CtS_2_*BU_2*2…_+CtS_2_*CtS_23_*BU_2*23…_+CtS_23_*CtS_23_*BU_23*23_

C_NonBCRU_=B_NonBCRU_+CtS_1_*NU_1_+CtS_2_*NU_2…_+CtS_23_*NU_23_+CtS_1_*CtS_1_*NU_1*1_+CtS_1_*CtS_2_*NU_1*2…_+CtS_1_*CtS_23_*NU_1*23_+CtS_2_*CtS_2_*NU_2*2…_+CtS_2_*CtS_23_*NU_2*23…_+CtS_23_*CtS_23_*NU_23*23_

BCR Urine P Score=C_BCRU_-C_NonBCRU_

Whereas A_BCRU_ is BCR constant, B_NonBCRU_ is Non-BCR constant, CtS_1_ through CtS_23_ are CtS values of gene 1 through gene 23, BU_1_ through BU_23_ are BCR regression coefficients of gene 1 through gene 23, BU_1*1_ through BU_23*23_ are gene 1 and gene 1 cross BCR regression coefficients through gene 23 and gene 23 cross BCR regression coefficients, NU_1_ through NU_23_ are Non-BCR regression coefficients of gene 1 through gene 23, and NU_1*1_ through NU_23*23_ are gene 1 and gene 1 cross Non-BCR regression coefficients through gene 23 and gene 23 cross Non-BCR regression coefficients. The sample was predicted to have BCR when BCR Urine P Score was >0, whereas the sample was predicted to have no BCR (Non-BCR) when BCR Urine P Score was ≤0.

For distant metastatic cancer prediction in the urine samples, the relative Ct (CtS) values of the 23 genes in the classifier were used to generate a classification score (MET Urine P Score) for each sample using the MET Urine Prediction Algorithm as shown below:

C_METU_=A_METU_+CtS_1_*MU_1_+CtS_2_*MU_2…_+CtS_23_*MU_23_+CtS_1_*CtS_1_*MU_1*1_+CtS_1_*CtS_2_*MU_1*2…_+CtS_1_*CtS_23_*MU_1*23_+CtS_2_*CtS_2_*MU_2*2…_+CtS_2_*CtS_23_*MU_2*23…_+CtS_23_*CtS_23_*MU_23*23_

C_NonMETU_=B_NonMETU_+CtS_1_*NMU_1_+CtS_2_*NMU_2…_+CtS_23_*NMU_23_+CtS_1_*CtS_1_*NMU_1*1_+CtS_1_*CtS_2_*NMU_1*2…_+CtS_1_*CtS_23_*NMU_1*23_+CtS_2_*CtS_2_*NMU_2*2…_+CtS_2_*CtS_23_*NMU_2*23…_+CtS_23_*CtS_23_*NMU_23*23_

MET Urine Prediction Score=C_METU_-C_NonMETU_

Whereas A_METU_ is metastasis constant, B_NonMETU_ is Non-metastasis constant, CtS_1_ through CtS_23_ are CtS values of gene 1 through gene 23, MU_1_ through MU_23_ are metastasis regression coefficients of gene 1 through gene 23, MU_1*1_ through MU_23*23_ are gene 1 and gene 1 cross metastasis regression coefficients through gene 23 and gene 23 cross metastasis regression coefficients, NMU_1_ through NMU_23_ are Non-metastasis regression coefficients of gene 1 through gene 23, and NMU_1*1_ through NMU_23*23_ are gene 1 and gene 1 cross Non-metastasis regression coefficients through gene 23 and gene 23 cross Non-metastasis regression coefficients. The sample was predicted to have distant metastatic cancer (MET) when MET Urine Prediction Score was >0, whereas the sample was predicted to have no distant metastatic cancer (Non-MET) when MET Urine Prediction Score was ≤0.

**Prostate tissue cohort and sample diagnosis**

The MSKCC prostate tissue specimen cohort used in the study consisted of dataset obtained from MSKCC Prostate Oncogenome Project at cBioPortal ([www.cbioportal.com](http://www.cbioportal.com)) database.^15^ The transcriptome profiles of PCa tissue specimens from 218 patients were included in the MSKCC dataset. Total RNA was purified from dissected prostate tissue specimens with at least 70% tumor cell content using TRIzol@Reagent Total RNA Isolation Reagent (Invitrogen, [Carlsbad, CA](https://www.google.com/search?q=Carlsbad,+California&stick=H4sIAAAAAAAAAOPgE-LUz9U3MDNLKUxS4gAxi0zK87S0spOt9POL0hPzMqsSSzLz81A4VhmpiSmFpYlFJalFxYtYRZwTi3KKkxJTdBScE3My0_KL8jITARf5WdBaAAAA&sa=X&ved=2ahUKEwiAleCJzuPkAhULWK0KHYJsDWwQmxMoATAeegQIDRAH), USA). The exon and whole-transcript expression levels were determined by using Affymetrix Human Exon 1.0 ST arrays.^15^ Aroma Affymetrix was used to process raw CEL files by standard RMA background adjustment and expression data normalization.^15^ The quantitative mRNA expression Z-Scores of the 23 genes in the classifier were obtained from the dataset at cBioPortal along with patients’ clinicopathological information, such as pre-operative PSA, Gleason score, and occurrence of BCR after RP or distant cancer metastasis during the follow-up period. After excluding patients with no Z-Score of the genes in the classifier or no clinicopathological information, the MSKCC cohort was acquired with 140 patients.

For BCR prediction in the prostate tissue specimen cohort MSKCC, the mRNA expression Z-Scores of the 23 genes in the classifier were used to generate a classification score (BCR Tissue P Score) for each specimen using the BCR Tissue Prediction Algorithm as shown below:

C_BCRT_=A_BCRT_+ZS_1_*BT_1_+ZS_2_*BT_2…_+ZS_23_*BT_23_+ZS_1_*ZS_1_*BT_1*1_+ZS_1_*ZS_2_*BT_1*2…_+ZS_1_*ZS_23_*BT_1*23_+ZS_2_*ZS_2_*BT_2*2…_+ZS_2_*ZS_23_*BT_2*23…_+ZS_23_*ZS_23_*BT_23*23_

C_NonBCRT_=B_NonBCRT_+ZS_1_*NT_1_+ZS_2_*NT_2…_+ZS_23_*NT_23_+ZS_1_*ZS_1_*NT_1*1_+ZS_1_*ZS_2_*NT_1*2…_+ZS_1_*ZS_23_*NT_1*23_+ZS_2_*ZS_2_*NT_2*2…_+ZS_2_*ZS_23_*NT_2*23…_+ZS_23_*ZS_23_*NT_23*23_

BCR Tissue P Score=C_BCRT_-C_NonBCRT_

Whereas A_BCRT_ is BCR constant, B_NonBCRT_ is Non-BCR constant, ZS_1_ through ZS_23_ are mRNA expression Z-Score of gene 1 through gene 23, BT_1_ through BT_23_ are BCR regression coefficients of gene 1 through gene 23, BT_1*1_ through BT_23*23_ are gene 1 and gene 1 cross BCR regression coefficients through gene 23 and gene 23 cross BCR regression coefficients, NT_1_ through NT_23_ are Non-BCR regression coefficients of gene 1 through gene 23, and NT_1*1_ through NT_23*23_ are gene 1 and gene 1 cross Non-BCR regression coefficients through gene 23 and gene 23 cross Non-BCR regression coefficients. The specimen was predicted to have BCR when BCR Tissue P Score was >0, whereas the specimen was predicted to not have BCR (Non-BCR) when BCR Tissue P Score was ≤0.

**Statistical analysis**

Univariate and multivariate Cox regression analysis and Kaplan Meier survival plot of BCR-free survival for the 23-Gene Classifier as well as cancer stage and Gleason score were conducted using SPSS (IBM, Armonk, New York). To assess BCR predictive accuracy of the 23-Gene Classifier, the classification of BCR or non-BCR of the samples by the 23-Gene Classifier BCR Prediction Algorithm was compared with the clinical diagnosis of BCR or non-BCR during follow-up to calculate sensitivity, specificity, positive predictive value (PPV), negative predictive value (NPV), and their respective 95% confidence intervals (CI) using discriminant analysis (DA) in XLSTAT (Addinsoft Inc, New York, NY). The receiver operating characteristic (ROC) curve was plotted and the area under the curve (AUC) with 95% CI was calculated. For further validation, the leave-one-out cross-validation analysis was performed by computing the prediction for a given sample if it was left out of the estimation sample and the process was repeated until all of the samples were left out once using XLSTAT. The regression coefficients generated by cross-validation test were used to classify each sample, which was then compared with the clinical diagnosis of each sample to calculate the diagnostic performance of cross-validation. Gleason score was used to classify each sample as having BCR or non-BCR by DA and such classification by Gleason score was then compared with the clinical diagnosis of BCR or non-BCR during follow-up to calculate sensitivity, specificity, PPV, NPV, and their respective 95% CI for Gleason score. Similarly, cancer stage was used to classify each sample as having BCR or non-BCR using DA and such classification by cancer stage was then compared with the clinical diagnosis of BCR or non-BCR during follow-up to calculate sensitivity, specificity, PPV, NPV, and their respective 95% CI for PSA. ROC curves were plotted for Gleason score and cancer stage, and their respective AUC with 95% CI were calculated. To compare the predictive performance of the 23-Gene Classifier with Gleason score and cancer stage, univariate and multivariate logistic regression analyses were performed. To assess accuracy of the 23-Gene Classifier for prediction of distant cancer metastasis, the metastatic or non-metastatic cancer classification of the samples by the 23-Gene Classifier MET Prediction Algorithm was compared with the clinical diagnosis of metastatic or non-metastatic cancer during follow-up to calculate sensitivity, specificity, PPV, NPV, AUC and their respective 95% CI. Similarly, the accuracy of Gleason score and combining the 23-Gene Classifier with Gleason score for prediction of metastatic cancer was assessed by DA analysis.
